# Smartphone App–Based Contingency Management and Opioid Use Disorder Treatment Outcomes

**DOI:** 10.1001/jamanetworkopen.2024.48405

**Published:** 2024-12-02

**Authors:** Elise N. Marino, Tara Karns-Wright, Matthew C. Perez, Jennifer S. Potter

**Affiliations:** 1 Be Well Institute on Substance Use and Related Disorders, Department of Psychiatry and Behavioral Sciences, University of Texas Health Science Center at San Antonio; 2 Be Well Institute on Substance Use and Related Disorders, University of Texas Health Science Center at San Antonio

## Abstract

**Importance:**

Opioid use disorder continues to be a national crisis, contributing to substantial morbidity and mortality. Medication is the only evidence-based treatment for opioid use disorder; however, improving clinical outcomes may require augmenting it with psychosocial interventions, such as contingency management (CM), specifically, smartphone app–based CM.

**Objective:**

To evaluate whether augmenting medication for opioid use disorder (MOUD) with app-based CM is associated with fewer days of opioid use at the end of treatment and greater retention than treatment with MOUD only.

**Design, Setting, and Participants:**

This retrospective cohort study used data from November 1, 2020, to November 30, 2023, collected from opioid treatment programs and office-based opioid treatment programs across Texas. The cohort included individuals aged 18 years or older who were uninsured or underinsured and who chose to receive MOUD only or MOUD plus CM delivered via the WEconnect smartphone app. Participants who chose to receive app-based CM received monetary incentives upon achieving their self-chosen recovery goals.

**Exposure:**

Opioid use disorder.

**Main Outcomes and Measures:**

Primary analyses examined differences in treatment outcomes (self-reported days of opioid use at the end of treatment and retention) between patients who chose MOUD only and those who chose MOUD plus app-based CM. Linear regression analysis was used to examine days of opioid use at the end of treatment, and Cox proportional hazards regression analysis was used to examine retention.

**Results:**

The analytic sample comprised 600 individuals (mean [SD] age, 38.4 [8.6] years; 342 male [57.0%]), with 300 included in each treatment group. Those who chose to receive MOUD plus app-based CM reported significantly fewer days of opioid use at the end of treatment compared with those who chose to receive MOUD only (mean [SD] duration, 8.4 [12.9] vs 12.0 [13.5] days; β = −6.10; 95% CI, −8.09 to −4.10). Retention analysis showed that patients who chose to receive MOUD plus app-based CM were significantly more likely to stay in treatment longer compared with those who chose to receive MOUD only (mean [SD] duration, 290.2 [109.4] vs 236.1 [128.1] days; β = 51.91; 95% CI, 33.86 to 69.95).

**Conclusions and Relevance:**

These findings suggest that augmenting MOUD with app-based CM may provide clinical benefits for underserved patients. Expanding the availability of app-based CM may contribute to decreasing the societal, economic, and personal burden of opioid use.

## Key Points

**Question:**

What is the association between using smartphone app–based contingency management with medication for opioid use disorder (MOUD) and treatment outcomes?

**Findings:**

In this cohort study of 600 individuals treated for opioid use disorder, those who chose to use app-based contingency management with MOUD reported fewer days of opioid use at the end of treatment and stayed in treatment longer than those who chose to only use MOUD.

**Meaning:**

These findings suggest that adding app-based contingency management to MOUD is associated with better treatment outcomes in clinical settings.

## Teaser

This cohort study compares treatment outcomes associated with augmented medication for opioid use disorder with smartphone app–based contingency management vs medication only.

## Introduction

In 2018, the annual societal cost related to opioid use disorder in the US was $968.9 billion.^1^ Medication for opioid use disorder (MOUD), including methadone, buprenorphine, and naltrexone, is the only evidence-based treatment for opioid use disorder. This first-line treatment has been shown to significantly decrease opioid-related morbidity and mortality,^2,3,4^ with consistent cost-saving benefits.^5^ Although MOUD is often effective at reducing personal and societal harms, some patients still experience difficulties with continued opioid use and adherence to treatment.^6,7^ Adequately treating individuals with opioid use disorder necessitates taking a multipronged approach, such as augmenting MOUD for optimal outcomes.

Many conditions benefit from dual treatment (ie, medication and therapy). One therapy for opioid use disorder is contingency management (CM), which provides financial incentives for accomplishing treatment goals. This therapy has traditionally been delivered in clinics using an opioid-negative finding on urine drug screens as the goal. While there are mixed findings regarding the benefits of adding CM to MOUD,^8^ several reviews and studies have found that individuals treated with MOUD plus CM had better retention and fewer opioid-positive findings on urine drug screens,^8,9,10,11^ suggesting that this combination may be effective for some. Although many individuals benefit from adding CM to MOUD, a long-standing limitation of this treatment is the requirement to attend multiple in-person appointments per week. With the recent COVID-19 pandemic, many patients were unwilling to attend in-person CM visits due to fear of contracting the virus.^12^ Additional factors have supported making telehealth and leveraging other technologies more common because they overcome access barriers, such as transportation, distance from the clinic, and childcare, that make attending in-person visits difficult.

Contingency management can now be delivered virtually using smartphone apps for substance use disorders^13,14,15,16,17,18,19,20,21^ and specifically opioid use disorder.^22,23,24^ While app-based CM has been associated with improved treatment outcomes, additional work is needed to understand the feasibility and effectiveness for opioid use disorder. In the modest research that exists, this treatment combination has been shown to increase buprenorphine adherence and decrease health care costs, emergency department visits, and inpatient admissions,^24^ suggesting that MOUD plus app-based CM may be effective.

Retrospective analyses of existing data are a strategy to understand the effects of augmenting MOUD with app-based CM, specifically the WEconnect Health^25^ CM smartphone app. Unlike traditional CM, WEconnect delivers evidence-based CM embedded in a recovery-oriented framework. In addition to substance-related behavioral targets, it permits patients to set daily goals that are personally meaningful, both substance use related and not (eg, attend a Narcotics Anonymous meeting, go for a walk, read). The app also includes a platform for tracking patients’ progress and payment and provides encouragement for completing their daily goals. Additionally, WEconnect offers 1-to-1 peer support and online meetings facilitated by certified peers. In contrast to traditional CM, the app permits patients to make decisions regarding their own goals for treatment and to explore recovery through peer support services available anywhere and accessible outside normal clinic hours.

Our goal was to retrospectively compare differences in treatment outcomes (days of opioid use at the end of treatment and retention, as these are particularly relevant to opioid use disorder outcomes^26,27^) among clinically treated patients who chose to use MOUD only compared with those who used MOUD plus the CM app delivered as part of standard clinical care. We tested the following a priori hypotheses: (1) patients who chose to use app-based CM with MOUD would report fewer days of opioid use at the end of treatment compared with those who chose to use MOUD only, and (2) patients who chose to use app-based CM with MOUD would stay in treatment longer than those who chose to use MOUD only.

## Methods

### Patients and Procedures

This retrospective cohort study investigated treatment outcomes among patients who were receiving MOUD from an opioid treatment program (OTP) or an office-based opioid treatment (OBOT) program in a publicly funded statewide network across Texas from November 1, 2020, to November 30, 2023. Because data were collected as part of standard clinical care, the University of Texas Health Science Center at San Antonio Institutional Review Board determined that this study was exempt from review, and patients were not required to provide informed consent. The article follows the Strengthening the Reporting of Observational Studies in Epidemiology (STROBE) reporting guideline for observational cohort studies.

Opioid treatment programs must provide comprehensive treatment services as regulated by 42 CFR 8, Medications for the Treatment of Opioid Use Disorder,^28^ including MOUD (methadone, buprenorphine, or naltrexone), physical examinations, counseling, case management, health education, and other supportive services (eg, treatment for co-occurring mental health conditions). Medications for opioid use disorder are administered directly to patients on site, with limitations on take-home doses. Office-based opioid treatment programs provide outpatient services that may occur in a substance use clinic, in a private medical setting, through telehealth, or in another type of practice setting. Unlike methadone MOUD, there is no federal oversight of the OBOT itself; the clinician is accountable to the state licensing board.

The statewide network (Be Well Texas) is funded by the Texas Targeted Opioid Response program at the Texas Health and Human Services Commission (HHSC) through contractual agreement with the University of Texas Health Science Center. The network was established to procure and implement MOUD services and enhance access to MOUD in Texas for individuals who are uninsured or underinsured, including those residing in historically underserved remote or rural areas. Patients receiving care in the network must be Texas residents who are at least 18 years old, have a diagnosis of opioid use disorder, and meet financial criteria for HHSC-funded substance use disorder services. Individuals who are uninsured or underinsured and who meet the eligibility requirements via HHSC’s sliding fee scale, which is determined based on family size and income relative to the poverty line, may have their cost for treatment fully or partially subsidized.

As part of the program design, CM was identified to support adherence. Smartphone technologies ensure that CM services are feasible, compliant, and delivered consistently. Digital CM provides administrative support to clinicians otherwise unable or unwilling to implement the approach. All OTPs and OBOT programs in the network were provided the opportunity to participate in the WEconnect program, and clinicians were responsible for implementation, with onboarding support from WEconnect. WEconnect memberships were offered to patients who chose to participate. Enrollment was not required and had no consequences regarding their ability to receive MOUD. WEconnect was available throughout the study period. All clinicians received an orientation to the app^25^ and had access to technical assistance from the app developer throughout the study period. Of the 23 programs (5 OTP, 18 OBOT) that were contracted as part of the network, 20 onboarded at least 1 patient to app-based CM (4 OTP, 16 OBOT). Patients were given access to the smartphone app for 1 year.

Our study included all patients who initiated MOUD in the network from November 1, 2020, to November 30, 2022. Follow-up continued through November 30, 2023, to allow all patients access to the full year of the WEconnect membership. Patients were not randomized into treatment groups because all were offered the opportunity to receive app-based CM as an adjunctive to their standard MOUD treatment. All patients received standard MOUD care for their OTP or OBOT program; those who chose to receive MOUD plus CM received standard care for their program plus app-based CM delivered via WEconnect Health.^25^

### WEconnect Health

The WEconnect smartphone app is a patient-driven, recovery-oriented CM intervention.^25^ In the WEconnect app, patients set daily goals of their own choosing that include both substance use and self-care (eg, take MOUD, attend treatment visit, exercise, talk to a friend). These serve as the treatment goals that are financially reinforced upon completion. WEconnect also includes a platform for tracking patient progress and payment and provides encouragement upon completion of their daily goals. Additionally, patients can engage in 1-to-1 peer support (ie, interactions with an individual who has lived experience with substance use and recovery) and join online support meetings that occur at least 8 times a day, 7 days a week.

Patients could earn up to $800 in incentives provided by WEconnect Health over the course of the year-long subscription. Incentives were in the form of digital gift cards redeemable immediately from approved retailers. Gift card offerings included clothing stores, bookstores, department stores, automotive accessories stores, grocery stores, home improvement stores, rideshare apps, a pharmacy, streaming service, restaurants, and office supply stores. There were no restrictions on what could be purchased if it was available by the retailer selected.

### Measures

Demographics included age, sex, and race (African American or Black, American Indian or Alaska Native, Asian, White, multiracial) and ethnicity (Hispanic or Latino, not Hispanic or Latino). These patient characteristics were recorded by the clinician but self-identified by the patient. Demographics were included because they were used in the statistical matching procedure.

Duration of opioid use at the end of treatment (in days) was captured at the patients’ last appointment before dropping out of treatment or at the 1-year end point of WEconnect using the substance use disorder assessment. This assessment, in which clinicians asked patients, “In the past 30 days, how many days have you used,” is a requirement for all patients. To examine retention, number of days in treatment was defined as the number of days the patient received MOUD care up to the 1-year end point (ie, 365 days). Patients were retained as long as they maintained an active prescription for MOUD and clinic contact.^29^ For buprenorphine, an active prescription and clinic contact within a 60-day period was required. For methadone, dosing and clinic contact within a 30-day period was required. These data were extracted from the electronic health record.

### Statistical Analysis

Patients who chose MOUD only represented a significantly larger group than those who chose MOUD plus app-based CM (3130 vs 622), and patients were not randomized into the 2 treatment groups. To create comparable groups for our analyses, we used matched control sampling using the entire sample of patients to create groups that were similar in size and characteristics. The matched dataset included an MOUD-only group matched to an MOUD plus app-based CM group with respect to the demographic characteristics of all individuals (age, sex, race, and ethnicity). The matched control variables were selected based on the association between our demographic variables and their influence on treatment access and outcomes. Using the nearest neighbor matching method, we estimated a propensity score that represented the probability of receiving our intervention for the selected covariates through a logistic regression. Each record in the intervention group was matched with a record in the control group based on closest proximity (evaluated based on the difference between propensity scores) for their propensity score without replacement. The MOUD-only group was taken from the same clinics as the MOUD plus app-based CM group. Table 1 shows prematch and postmatch participant characteristics. Matched control sampling is the optimal way to closely approximate randomized trials,^30^ and it increases statistical power while controlling for confounding factors.

**Table 1.** Prematch and Postmatch Participant Characteristics

Characteristic	No. of participants (%)		
	Prematch (N = 3759)	Postmatch	
		MOUD (n = 300)	MOUD + CM (n = 300)
Age, mean (SD), y	39.9 (9.2)	38.1 (8.6)	39.5 (9.0)
Sex			
Female	1872 (49.8)	138 (46.0)	120 (40.0)
Male	1887 (50.2)	162 (54.0)	180 (60.0)
Race			
African American or Black	210 (5.6)	9 (3.0)	9 (3.0)
American Indian or Alaska Native	20 (0.5)	1 (0.3)	1 (0.3)
Asian	12 (0.3)	1 (0.3)	1 (0.3)
White	3455 (91.9)	285 (95.0)	284 (94.7)
Multiracial	56 (1.5)	4 (1.3)	5 (1.7)
Ethnicity			
Hispanic or Latino	1424 (37.9)	86 (28.7)	107 (35.7)
Not Hispanic or Latino	2335 (62.1)	214 (71.3)	193 (64.3)
Treatment setting			
OTP	1867 (49.7)	187 (62.3)	124 (41.3)
OBOT	1892 (50.3)	113 (37.7)	176 (58.7)

Abbreviations: CM, contingency management; MOUD, medication for opioid use disorder; OBOT, office-based opioid treatment; OTP, opioid treatment program.

Bivariate analyses comparing demographic and clinical characteristics between the MOUD-only and MOUD plus app-based CM groups were conducted using χ^2^ tests for categorical variables and 2-tailed *t* tests for continuous variables. To examine differences in treatment outcomes (self-reported days of opioid use at the end of treatment, retention) between MOUD only and MOUD plus app-based CM, linear regression was used to examine duration of opioid use, and Cox proportional hazards regression was used to examine retention. Sensitivity analyses were then conducted to ensure that our findings were not merely a result of the matched control sampling by replicating the original models using the entire sample of patients. In the multivariable models, all variables were entered simultaneously. Reference groups included OBOT (treatment setting), buprenorphine only (MOUD), and MOUD only (treatment group). A 2-sided *P* < .05 was considered significant. The data analysis was performed using R, version 4.3.3 (R Project for Statistical Computing).

## Results

In the entire sample of 3759 patients, 1892 (50.3%) were from an OBOT program and 1867 (49.7%) from an OTP (Table 1). Overall, 622 patients (16.5%) used the app, 360 (57.9%) of whom were from an OBOT program and 262 (42.1%) from an OTP.

After the matched control sampling, 600 individuals were included in this analysis (mean [SD] age, 38.4 [8.6] years; 258 female [43.0%] and 342 male [57.0%]), of whom 300 belonged to the MOUD-only group and 300 the MOUD plus app-based CM group. The majority of the cohort included patients identifying as White (569 [94.8%] compared with 18 identifying as African American or Black [3.0%], 2 as American Indian or Alaska Native [0.3%], 2 as Asian [0.3%], and 9 as multiracial [1.5%]) and not Hispanic or Latino (407 [67.8%] compared with 193 identifying as Hispanic or Latino [32.2%]) (Table 2).

**Table 2.** Demographic and Clinical Characteristics

Characteristic	No. of participants (%)			*P* value
	Overall (N = 600)	MOUD (n = 300)	MOUD + CM (n = 300)	
Age, mean (SD), y	38.4 (8.6)	38.1 (8.6)	39.5 (9.0)	.71
Sex				
Female	258 (43.0)	138 (46.0)	120 (40.0)	.93
Male	342 (57.0)	162 (54.0)	180 (60.0)	
Race				
African American or Black	18 (3.0)	9 (3.0)	9 (3.0)	.97
American Indian or Alaska Native	2 (0.3)	1 (0.3)	1 (0.3)	
Asian	2 (0.3)	1 (0.3)	1 (0.3)	
White	569 (94.8)	285 (95.0)	284 (94.7)	
Multiracial	9 (1.5)	4 (1.3)	5 (1.7)	
Ethnicity				
Hispanic or Latino	193 (32.2)	86 (28.7)	107 (35.7)	.97
Not Hispanic or Latino	407 (67.8)	214 (71.3)	193 (64.3)	
Treatment setting				
OTP	311 (51.8)	187 (62.3)	124 (41.3)	<.001
OBOT	289 (48.2)	113 (37.7)	176 (58.7)	
MOUD^a^				
Buprenorphine only	315 (52.5)	135 (45.0)	180 (60.0)	<.001
Methadone only	276 (46.0)	160 (53.3)	116 (38.7)	
Retained in treatment	303 (50.5)	118 (39.3)	185 (61.7)	<.001
Duration of treatment, mean (SD), d	263.1 (122.1)	236.1 (128.1)	290.2 (109.4)	<.001
Duration of opioid use at end of treatment, mean (SD), d	10.2 (14.3)	12.0 (13.5)	8.4 (12.9)	<.001

Abbreviations: CM, contingency management; MOUD, medication for opioid use disorder; OBOT, office-based opioid treatment; OTP, opioid treatment program.

^a^ Six patients started treatment taking methadone and transitioned to buprenorphine, and 3 patients started treatment taking buprenorphine and transitioned to naltrexone.

Patients who chose MOUD plus app-based CM were significantly more likely to be in an OBOT program than an OTP (176 [58.7%] vs 124 [41.3%]; *P* < .001), consistent with the entire sample. There was also a significant difference in MOUD where patients who chose MOUD plus app-based CM were more likely to be taking buprenorphine than methadone (180 [60.0%] vs 116 [38.7%]; *P* < .001). Overall, 185 patients (61.7%) in the MOUD plus app-based CM group were retained in treatment for the full year, and 118 patients (39.3%) in the MOUD-only group were retained (*P* < .001) (Table 2).

Because there were significant differences in treatment setting and MOUD between the 2 groups, both were controlled for in multivariable analyses. Patients who chose MOUD plus app-based CM reported significantly fewer days of opioid use at the end of treatment compared with those who chose MOUD only (mean [SD] duration, 8.4 [12.9] vs 12.0 [13.5] days; β = −6.10; 95% CI, −8.09 to −4.10; *P* < .001) (Table 2 and Table 3). Furthermore, patients who chose MOUD plus app-based CM were significantly more likely to stay in treatment longer compared with those who chose MOUD only (mean [SD] duration, 290.2 [109.4] vs 236.1 [128.1] days; β = 51.91; 95% CI, 33.86 to 69.95; *P* < .001). Sensitivity analyses were run with the entire sample, and all results remained the same (eTable in Supplement 1).

**Table 3.** Augmenting MOUD With Smartphone App–Based CM Treatment Outcomes

Outcome	β (95% CI)	*P* value
**Days of opioid use at end of treatment** ^a^		
Treatment setting	1.56 (−2.81 to 5.93)	.48
MOUD		
Buprenorphine to naltrexone	5.53 (−8.29 to 19.35)	.43
Buprenorphine to methadone	9.15 (−0.29 to 18.59)	.06
Methadone only	0.60 (−3.80 to 5.00)	.79
Treatment group	−6.10 (−8.09 to −4.10)	<.001
**Retention** ^b^		
Treatment setting	38.95 (−0.58 to 78.48)	.053
MOUD		
Buprenorphine to naltrexone	−154.72 (−250.23 to −59.20)	.01
Buprenorphine to methadone	−67.11 (−140.02 to 5.80)	.07
Methadone only	35.58 (−4.32 to 75.48)	.08
Treatment group	51.91 (33.86 to 69.95)	<.001

Abbreviations: CM, contingency management; MOUD, medication for opioid use disorder.

^a^ Linear regression analysis.

^b^ Cox proportional hazards regression analysis. Reference groups: office-based opioid treatment (treatment setting), buprenorphine only (MOUD), and MOUD only (treatment group).

## Discussion

The findings from this retrospective cohort study confirm both a priori hypotheses that individuals who chose to use app-based CM with MOUD would report significantly fewer days of opioid use at the end of treatment and have significantly higher retention than those who chose MOUD only. To our knowledge, no other study has reported these findings among publicly funded patients who are underinsured or uninsured and who elected whether to use app-based CM with their MOUD.

Our results are further strengthened by the feasibility of adding recovery-oriented, app-based CM to MOUD in clinical practice. Others have also found that CM delivered via a smartphone app is feasible and associated with positive treatment outcomes; however, these prior studies used abstinence, confirmed via at-home drug testing (urine, saliva, breathalyzer, etc), as the treatment goal.^13,14,17,19,20,21,22,23,31^ In contrast to this traditional approach to app-based CM, our study included CM delivered via a smartphone app that used patient-chosen treatment goals in addition to recovery support tools. Specifically, the app provided financial reinforcement for completing treatment goals, as well as adjunctive support from peer specialists who were accessible both during and outside of normal clinic operating hours. Use of this novel approach to delivering an app-based intervention provides a more customized treatment experience through which patients feel involved in the decisions regarding their personal recovery journey, which increases engagement and satisfaction while at the same time produces positive treatment outcomes.^32^

One of the unique and potentially appealing features of the WEconnect app is the inclusion of treatment goals focused on substance use and recovery (eg, mental and physical health, family, friends, leisure) rather than a binary definition of treatment success. Augmenting MOUD with WEconnect was associated with better treatment outcomes; however, less than one-fifth of our entire sample chose to use it. Obtaining, owning, and navigating app-enabled devices may be barriers for some patients, and these apps require consistent use, which may become burdensome or unappealing over time, leading to low use. It is also possible that clinicians themselves may benefit from additional education and training to support adoption.^33^ Considering the potential benefits of WEconnect and the challenges of engaging patients in app-based interventions, our findings indicate that self-selection into this treatment may be an important factor for both use and treatment outcomes. In line with the framework of current research of the National Institute on Drug Abuse Clinical Trials Network,^34,35^ using our study to inform a randomized effectiveness or implementation trial may help to elucidate how we can increase both the benefits and use of this app-based intervention.

As a virtual treatment, app-based CM has fewer infrastructure barriers to implementation and should provide opportunities for rapid dissemination to patients; however, there remain barriers to achieving this that must be challenged. Despite its observed effectiveness, CM has shown a slow uptake in clinical practice, in part related to concerns regarding the added cost of using CM, especially as an adjunctive treatment. Clinician attitudes may also play a role in adoption. Yet, new research has found that among all treatments for opioid use disorder, CM plus MOUD (specifically methadone) is associated with the greatest cost-saving benefits.^36^ Expanding the availability of CM, including this emerging app-based CM, has the potential to decrease the immense societal, economic, and personal burden of opioid use.

### Limitations

This study has some limitations worth noting. Because app-based CM was offered as part of standard clinical care, patients were not randomized to a treatment condition. There may also be selection bias for patients who received app-based CM and site differences within the OTPs and OBOT programs. For example, some clinicians may have more successfully introduced, explained, and engaged patients to accept the app. Additionally, most of the sample identified as White, which may limit the generalizability of these findings to other races and ethnicities. Despite these caveats, this study illuminates the potential benefits of using a virtual adaptation of an effective psychosocial intervention uniquely housed within a recovery framework.

## Conclusions

In this retrospective cohort study of augmenting MOUD, adding recovery-oriented CM delivered via a smartphone app was associated with less opioid use and greater treatment retention. These results are promising, and they highlight the potential importance of a patient’s decision to use app-based CM. Despite the challenges of engaging patients in other app-based interventions, adding recovery-oriented, app-based CM may be one way to enhance clinical care and meet the growing needs of historically underserved patients taking MOUD.

## Notes

Supplement 1. **eTable.** Sensitivity Analyses With the Entire Sample (N = 3759)

Supplement 2.
Data Sharing Statement
