# Supplementary material for: Smartphone App–Based Contingency Management and Opioid Use Disorder Treatment Outcomes
Source: JAMA Netw Open. 2024 Dec 2;7(12):e2448405. doi: 10.1001/jamanetworkopen.2024.48405 (PMC11612830; doi:10.1001/jamanetworkopen.2024.48405)
Supplement: Supplement 1. — eTable. Sensitivity Analyses With the Entire Sample (N = 3759) [file jamanetwopen-e2448405-s001.pdf]

Supplemental Online Content

Marino EN, Karns-Wright T, Perez MC, Potter JS. Smartphone app–based contingency management and opioid use disorder treatment outcomes. *JAMA Netw Open*. 2024;7(12):e2448405. doi:10.1001/jamanetworkopen.2024.48405

eTable. Sensitivity Analyses With the Entire Sample (N = 3759)

This supplemental material has been provided by the authors to give readers additional information about their work.

© 2024 Marino EN et al. *JAMA Network Open.*

eTable. Sensitivity Analyses With the Entire Sample (N = 3759)

**Days of Opioid Use at End of Treatment [a]**	**β estimate [95% Confidence Interval]**	***p* value**
Treatment setting	1.84 [-0.57, 4.24]	.13
MOUD		
Buprenorphine to Naltrexone	1.35 [-7.09, 9.79]	.75
Buprenorphine to Methadone	-2.43 [-6.64, 1.78]	.26
Methadone Only	-1.83 [-6.21, 2.55]	.41
Treatment group	-3.31 [-4.45, -1.85]	< .001
**Retention [b]**		
Treatment setting	38.84 [-0.68, 78.36]	.054
MOUD		
Buprenorphine to Naltrexone	-87.50 [-206.01, 31.01]	.15
Buprenorphine to Methadone	67.10 [-5.79, 139.99]	.07
Methadone Only	103.08 [29.79, 176.36]	.006
Treatment group	51.64 [33.61, 69.67]	< .001

[a] Linear regression analysis, [b] cox regression analysis. Reference groups: OBOT (treatment setting), buprenorphine only (MOUD), and MOUD only (treatment group).

© 2024 Marino EN et al. *JAMA Network Open.*
