# Supplementary material for: Smartphone App–Based Contingency Management and Opioid Use Disorder Treatment Outcomes
Source: JAMA Netw Open. 2024 Dec 2;7(12):e2448405. doi: 10.1001/jamanetworkopen.2024.48405 (PMC11612830; doi:10.1001/jamanetworkopen.2024.48405)
Supplement: Supplement 2. — Data Sharing Statement [file jamanetwopen-e2448405-s002.pdf]

# Data Sharing Statement

Marino. Smartphone App–Based Contingency Management and Opioid Use Disorder Treatment Outcomes. *JAMA Netw Open*. Published December 02, 2024. doi:10.1001/jamanetworkopen.2024.48405

**Data**
**Data available:** No
